# Supplementary material for: Prevalence of Schistosoma bovis and Schistosoma haematobium hybrids in endemic communities in Ghana
Source: PLoS One. 2026 Jan 7;21(1):e0339722. doi: 10.1371/journal.pone.0339722 (PMC12779034; doi:10.1371/journal.pone.0339722)
Supplement: S1 Table — (DOCX) [file pone.0339722.s001.docx]

**S1 Table: Characteristics of primers used in the characterisation PCR assay**

| **Species target** | **Sequence (5’-3’)** | **Amplicon size (bp)** | **Annealing Temperature**  **(°C)** | **Ref** |
| --- | --- | --- | --- | --- |
| *S. haematobium* | TGGTCATCCTGAGGTGTAT | 539 | 58.7 | Webster *et al*., 2009 |
|  | TGATAATCAATGACCCTGCAATAA |  |  |  |
| *S. bovis* | TGGGCATCCTGAGGTGTAT | 306 | 58.7 | Webster *et al*., 2009 |
|  | CACAGGATCAGACAAACGAGTACC |  |  |  |
