# Supplementary figures and images for: Prevalence of Schistosoma bovis and Schistosoma haematobium hybrids in endemic communities in Ghana
Source: PLoS One. 2026 Jan 7;21(1):e0339722. doi: 10.1371/journal.pone.0339722 (PMC12779034; doi:10.1371/journal.pone.0339722)

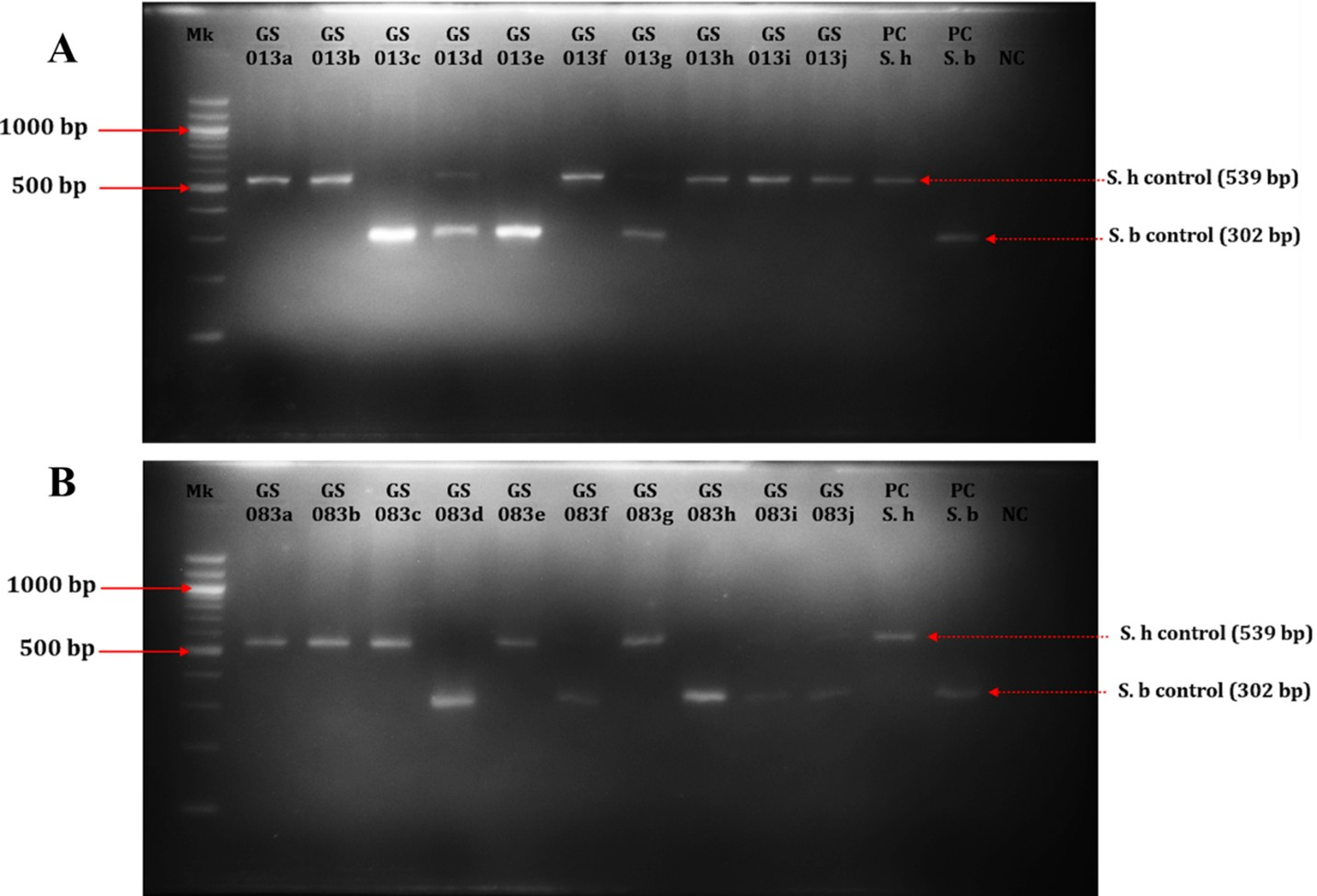

Supplement: S1 Fig — Lane 1–10 are loads of respective S. haematobium and S. bovis amplications as well as mix S. haematobium/S. bovis amplification in 10 single eggs from a participants’ DNA sample using the primers in S1 Table. Mk represents the molecular marker; PC is the positive control, and NC is the negative control. (TIFF) [file pone.0339722.s002.tiff]

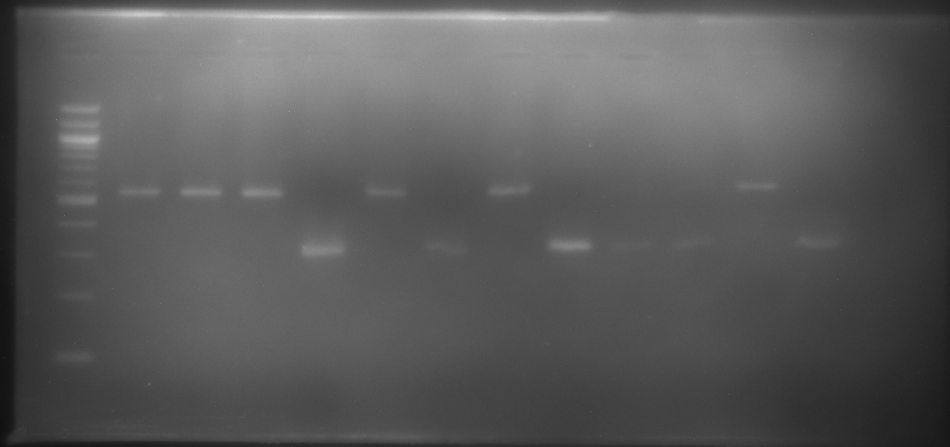

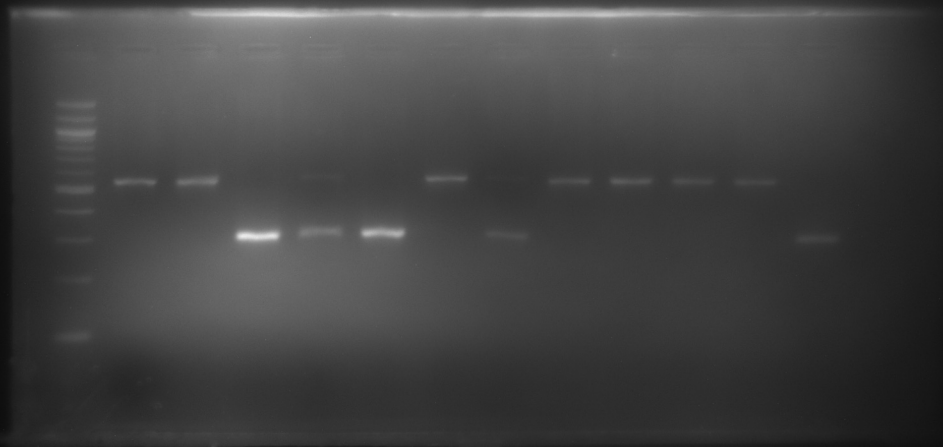

Supplement: S1 Raw Image — (PDF) [file pone.0339722.s003.pdf]
