# Supplementary material for: Prevalence of Schistosoma bovis and Schistosoma haematobium hybrids in endemic communities in Ghana
Source: PLoS One. 2026 Jan 7;21(1):e0339722. doi: 10.1371/journal.pone.0339722 (PMC12779034; doi:10.1371/journal.pone.0339722)
Supplement: Attachment — Submitted filename: Comment.docx [file pone.0339722.s004.docx]

Prevalence and Genetic Diversity of Schistosoma bovis and Schistosoma haematobium Hybrids in Endemic Communities in Ghana

Comments

Title

- Short and concise

Abstract

- Well written with all necessary information

Background

- Well written with the necessary information

Method section

- Better to include study area map

Study population

- be consistent with SAC rather than community
- Line 117 Cochran’s sample size formula was used in calculating for the total participants to be enrolled into the study. Using 118 a schistosomiasis prevalence of 44.2% reported in Ga South district (Cunningham et al., 2020)

Correct it as “Cochran’s sample size formula was used in calculating for the total participants to be enrolled into the study using ……..”

- Line 125 value inserted for P contains letter “e” ….

Line 132. In order to avoid confusion, it is better to indicate number of schools where 140 SAC are recruited

Ethical considerations

- Protocols are considered but the treatment of Schistosoma positive children is not indicated

PCR amplification

Whether the PCR reaction is multiplex or not is not

- Line 174-180 should be supported with reference

Statistical method

- Well described and are appropriate

Result

All necessary data are in place

Discussion

- Clear and sufficient

Conclusion

- Stems from the research findings

References

- Most are recent and hence acceptable
- Fig 1,2,&3 are repeated
