# Supplementary material for: Prevalence of Schistosoma bovis and Schistosoma haematobium hybrids in endemic communities in Ghana
Source: PLoS One. 2026 Jan 7;21(1):e0339722. doi: 10.1371/journal.pone.0339722 (PMC12779034; doi:10.1371/journal.pone.0339722)
Supplement: Attachment — Submitted filename: Responses to Reviewers Comments.docx [file pone.0339722.s005.docx]

Reviewer #1: This article reports the results of a cross-sectional study investigating the prevalence of Schistosoma sp. in schoolchildren from two communities in Ghana. The authors find regional differences in the prevalence of the parasite. Additionally, they demonstrate that two species, *S. haematobium* and *S. bovis*, occur—sometimes even in the same individual—which could lead to hybridization. Overall, the study is well-written and investigates an important topic of high relevance for public health. However, a few aspects are not well described, and there are some errors in the discussion, which can be addressed in a revision.

Major Comments

1. All sequenced samples need to be made publicly accessible.

**Response: We acknowledge the importance of data accessibility and transparency. All sequenced samples will be deposited in a public repository such as GenBank.**

2. Table 2 and Figure 1 refer to S. haematobium infection. This is incorrect since it is later shown that some of the infections are S. bovis or mixed amplifications. Perhaps the authors refer to samples visually identified as S. haematobium?

**Response: We appreciate the observation. In Table 2 and Figure 1, we referred to samples visually identified as *S. haematobium* based on microscopy prior to molecular analysis. We have revised the text to specify that initial identification was based on morphological assessment, with molecular analysis later revealing S. bovis and mixed infections.**

3. Figure 2 is never referred to in the text.

**Response: Thank you for pointing this out. Figure 2 has been appropriately cited on line 252 in the results section.**

4. It is unclear where the S. mansoni samples in Figure S1 and those "used as control" in Figure 3 came from.

**Response: The tree has been revised to exclude the *S. mansoni* samples. Only reference sequences for *S. mansoni* have been included in the development of the tree.**

5. The rooting of the tree in Figure 3 is unclear. The tree should either be rooted with an appropriate outgroup or displayed as an unrooted tree. Furthermore, samples M3, M5, and M10 are highly diverged from the other S. bovis samples. The discussion on this point is vague and ad hoc, referencing adaptation. However, alternative explanations should also be considered. For example, could these sequences represent another Schistosoma species? Are there any sequences on NCBI similar to these samples?

**Thank you for your valuable feedback. In the revised phylogenetic tree, we have ensured appropriate rooting and clarified the divergence of M3, M5, and M10. These samples form a strongly supported clade (bootstrap values 94–98) that is distinctly separated from other S. bovis sequences. While their divergence may suggest local adaptation, we acknowledge alternative possibilities, including cryptic species, hybridization, or taxonomic misclassification. To further investigate this, we have performed a BLAST search against NCBI databases and analysed the sequences to confirm their phylogenetic placement. We have integrated these findings into the revised discussion**.

Discussion has been revised to read:

**“The significant genetic divergence observed in the *S. bovis* samples M3, M5, and M10 suggests the presence of cryptic genetic diversity within the population in Ghana, potentially resulting from geographic isolation or ecological niche differentiation. Beyond environmental adaptation, alternative explanations such as hybridization and introgression events with *S. haematobium* or other *Schistosoma* species could account for their distinct genetic profiles. These samples may have undergone genetic recombination or backcrossing, leading to their differentiation from other *S. bovis* isolates. Additionally, host adaptation pressures, including variations in definitive hosts and immune responses, could have driven the accumulation of unique genetic mutations. Genetic drift and founder effects might also contribute to their divergence if they originated from isolated populations with limited genetic exchange. These findings emphasize the complexity of S. bovis population structure in Ghana and highlight the need for further genomic studies to better understand the evolutionary mechanisms at play.**

6. The statistical interpretation of the results requires careful revision. For example:

o In the discussion, it is stated: “The intensity of infection also varied significantly by age and gender.” However, in the results, it is stated: “No significant differences in the intensity of infection were observed across age groups or between genders in either study area [p > 0.05].”

**We acknowledge the discrepancies and have revised the discussion to ensure alignment with the results. The statement has been revised to read “Although intensity of infection did not vary significantly by age and gender, The intensity of infection also varied significantly by age and gender. Notably, higher intensity infections were observed among older age groups (13-17 years) in Ga South…….”**

o Similarly, it is stated that: “Gender differences in prevalence were also noted, with males showing higher infection rates in both districts.” However, Table 2 indicates no significant difference. The discussion must be corrected to accurately reflect the results.

**The statement has been revised on line 309 – 310 to read “Gender differences in prevalence were observed, with males exhibiting higher infection rates in both districts; however, the differences were not statistically significant”**

Minor Comments

• The abbreviation SAC (presumably school-aged children) is used only twice in the methods and is not introduced. It should either be explained or removed from the manuscript.

**The abbreviation has been explained on Line 130**

• Line 132: How were the schools chosen?

**Schools were selected based on information provided from the Ghana Health Service NTD programme on schools involved in PZQ MDA in the study districts. District SHEP officers aided in the selection of schools within the district. Also, schools were selected from communities with proximity to freshwater bodies. Three schools were selected in each district**

• Line 133: Does “140 children per school” mean per school or per community? Are school and community the same? Please elaborate.

**140 children per school per community within the district. Three schools were selected from three communities per district.**

• Line 162: Aliquots of ddH2O? Please clarify.

**The statement has been revised**

• Line 236: Before discussing the intensity of infection in different subgroups, it would be helpful to provide the overall proportions for each community. If the authors consider it relevant, they could also test whether the prevalence of severe infection is lower in Birim North (as this is likely).

**The discussion of the overall proportions for each community has been included in the revised manuscript on line 307 - 309**

• Line 269: This is confusing. Forty participant specimens × 10 samples each would yield 400 sequences, but there are fewer. Please explain in more detail how the samples were selected.

**A total of 30 specimens were selected,** **with 10 samples each representing distinct amplification profiles: S. haematobium (red), S. bovis (yellow), and both S. haematobium and S. bovis (blue). The total number of specimens corresponds to the unique amplification patterns observed, not to the number of individual samples per participant. This method was used to ensure representation of the different parasite strains in the analysis. This has been explained in section 2.5.3 of the methodology.**

• Figure 1: The visualizations could be simplified by using stacked bar plots within each age or gender category.

**Figure one has been revised to make use of stacked bar plots.**

• Figure 3: Include the legend for the colored symbols within the figure. Root the tree with an outgroup or display it as unrooted. Samples M5, M3, and M10 are likely a different species.

**Legend for Figure 3 has been updated with the coloured symbols within the figure as: K*: S. haematobium* (red); F: *S. haematobium/ S.bovis* (yellow); M: *S. bovis* (green). The tree has been rotted with F. hepatica as an out group. Samples M3 and M10 form a distinct monophyletic group suggesting genetic divergence from both parental species. Additionally, M5 clusters separately, exhibiting a closer phylogenetic relationship to *Fasciola hepatica* rather than the Schistosoma clade**

Reviewer #2: The authors had clearly defined the objectives of the study, followed appropriate study methodology and used appropriate statistical tools to describe their finding. The results are presented and discussed to show the findings corresponding the study objective.

Method section

Better to include study area map

Study population

be consistent with SAC rather than community

**Study Population has been revised accordingly**

· Line 117 Cochran’s sample size formula was used in calculating for the total participants to be enrolled into the study. Using 118 a schistosomiasis prevalence of 44.2% reported in Ga South district (Cunningham et al., 2020)

Correct it as “Cochran’s sample size formula was used in calculating for the total participants to be enrolled into the study using ……..”

**The statement has been revised as suggested**

· Line 125 value inserted for P contains letter “e” ….

**This has been addressed**

Line 132. In order to avoid confusion, it is better to indicate number of schools where 140 SAC are recruited

**The methodology has been revised**

Ethical considerations

· Protocols are considered but the treatment of Schistosoma positive children is not indicated

**The study did not treat positive participants. However, these schools are marked for annual PZQ MDA by the Ghana Health Service Neglected Tropical Disease Programme.**

PCR amplification

Whether the PCR reaction is multiplex or not is not

**The PCR was a multiplex reaction**

· Line 174-180 should be supported with reference

**The amplification process is an in-house optimised protocol. However, the primers used in the study have been appropriately referenced (Webster et al 2009).**

Reviewer #3: This study presents two major methodological problems that unfortunately cannot be circumvented.

- The first problem is that the authors used a mixture of parasites without even knowing the size of the mixture; it is therefore impossible to infer the hybrid character because pure genotype can be mixed. The only way to infer the hybrid character is to work on isolated individuals (i.e. miracidia) as it has been done in many previous studies. Figure 2, which shows prevalence by species, unfortunately makes no sense. How many parasites per patient are there to say that there are only Sh? or only Sb? We can imagine that the sample contains from 1 to several hundred individuals.

- The second problem is that the authors did not use any nuclear genes. Once again, it is impossible to give the hybrid character with only a mitochondrial gene. Numerous studies show the presence of S bovis mitochondria and an S bovis nuclear gene, too.

It's unfortunate, but from a molecular point of view, the only use of this study is to show that there are S bovis mitochondria in schistosomes from the urine of a patient from Ghana. No epidemiological dimension, or hypothesis on the hybrid character can be advanced. Finding S bovis mitochondria in patients' urine is very common in West Africa (shown in Senegal, Cameroon, Mali, Ivory Coast and Benin).

From a phylogenetic point of view, finding that patients with a Sh, Sb mixture have an intermediate position between Sb and Sh is an aberration. The authors forgot that mitochondria are heritable only from the mother's side, so there is no genetic cross: it's either Sb or Sh! - I'd be interested to see the chromatogram of a mixture of sequences from two species...

The authors use markers specific to Sb and Sh and use an S mansoni DNA as control; why do this if the primers are specific? The sites are sites where Sh and Sm are present (according to the authors); the authors should therefore also have checked for the presence of Sm genes (mitochondrial and nuclear), on an individual basis. Sm/Sh hybrids frequently exist

Recently, a publication accepted in the journal Am J Trop Med Hyg (1) using this same technique was the subject of a 'Letter to the Editor' (2) criticising this approach. I fully agree with the criticisms made. This methodological error must not be repeated (and not published), as it leads to misinterpretation.

(1) Enudi AO, Nmorsi OPG, Egwunyenga AO. Human Schistosomiasis due to Schistosoma bovis in Nigeria. Am J Trop Med Hyg. 2024 Oct 15;111(6):1230-1236. doi: 10.4269/ajtmh.23-0539. PMID: 39406250; PMCID: PMC11619502.

(2) Ajakaye OG, Enabulele EE. Schistosoma bovis Infecting Humans in Nigeria. Am J Trop Med Hyg. 2025 Jan 7:tpmd240714. doi: 10.4269/ajtmh.24-0714. Epub ahead of print. PMID: 39773834.

In conclusion, this article should not be published as it stands. The authors should publish epidemiological data only. The molecular epidemiology approach is not good.

**Thank you for your constructive feedback. We have addressed the concerns raised regarding the mixture of parasites and the inclusion of nuclear genes.**

**Regarding the concern about inferring hybridization in a mixture, we want to clarify that we used species-specific primers that do not cross-amplify between species. The use of single eggs from each pool of egg-positive samples for DNA extraction and analysis have been clarified in the methodology. This allowed us to accurately distinguish between the pure genotypes present in the sample. The primers ensured that we could separately amplify each species' mitochondrial DNA, and any potential hybrids would be clearly identified by the presence of both species-specific markers.**

**We acknowledge that mitochondrial data alone cannot confirm hybrid status. While our study focused on detecting S. haematobium and S. bovis mitochondrial sequences using species-specific primers, we do not claim hybridization based solely on these findings. However, our approach provides valuable insight into the presence of S. bovis mitochondria in S. haematobium-positive urine samples in Ghana. We agree that nuclear markers are necessary for definitive hybrid confirmation and plan to incorporate them in future studies.**

**We recognize that S. bovis mitochondrial sequences have been detected in several West African countries. Our findings contribute additional data from Ghana, reinforcing the widespread occurrence of S. bovis mitochondrial haplotypes in S. haematobium-endemic areas. While our study does not make direct epidemiological claims about hybridization, it highlights the need for further genetic investigations.**

**The phylogenetic tree was reconstructed using appropriate outgroups and reference sequences from GenBank. Since mitochondria are maternally inherited, the tree does not imply genetic recombination but rather illustrates mitochondrial haplotype diversity. The placement of certain sequences reflects mitochondrial lineage relationships rather than hybrid status.**

**The S. mansoni DNA control was included to validate the specificity of our S. haematobium and S. bovis-specific primers, particularly given the co-endemicity of S. mansoni in the study region. We agree that future studies should incorporate nuclear markers to assess the potential presence of S. mansoni/S. haematobium hybrids.**

**By using single eggs for DNA extraction, we have addressed concerns about mixed populations, providing clearer lineage identification. While our study does not claim to confirm hybridization, it demonstrates the presence of S. bovis mitochondria in S. haematobium-positive urine samples and emphasizes the need for further nuclear-based investigations. We appreciate the reviewer’s insights, which have strengthened the clarity and validity of our findings.**
