# Supplementary material for: Prevalence of Schistosoma bovis and Schistosoma haematobium hybrids in endemic communities in Ghana
Source: PLoS One. 2026 Jan 7;21(1):e0339722. doi: 10.1371/journal.pone.0339722 (PMC12779034; doi:10.1371/journal.pone.0339722)
Supplement: Attachment — Submitted filename: Response_to_Reviewer_Comments_auresp_3.docx [file pone.0339722.s007.docx]

Reviewer #4: The manuscript is well written and addresses a major public health concern. However, I have the following comments:

1. The authors should be careful to italicise scientific names.

**Response:**

We appreciate the reviewer’s observation. All the scientific names have been italicised.

2. The authors should define the meaning of 'light-' and 'high-intensity infections'.

**Response:**

We are grateful for this suggestion. *“The intensity of infection was categorised into negative, light intensity (1-49 eggs/10 mL), and heavy intensity (≥50 eggs/10 mL) based on WHO thresholds*” (lines 214-216).

3. While the authors mention the limitation of using just the COX1 in the analyses, it would be important for them to indicate what could be done differently in order to generate more robust phylogenetic results.

**Response:**

We appreciate the reviewer’s observation. We now specify that “*A more robust approach would include multilocus analyses, particularly the integration of both mitochondrial and nuclear markers, such as ITS, which would allow for better discrimination of parental lineages and hybrid genotypes. Whole-genome sequencing could also provide a higher level of resolution in identifying introgression patterns and clarifying evolutionary relationships.”* (Line 362 – 366).

Reviewer #5: To the authors,

The manuscript ‘Prevalence of Schistosoma bovis and Schistosoma haematobium Hybrids in Endemic Communities in Ghana’ (Manuscript Number: PONE-D-24-53141R2; Type: Research Article) presents some problems concerning both content and format.

Title

I consider that the title of this manuscript is ambitious (‘Prevalence of Schistosoma bovis’; ‘Schistosoma haematobium Hybrids’ is not fully addressed in the manuscript). The title could be either maintained (if the result & discussion address these aspects in detail) or be revised in order to better represent the body of the text.

Material and Methods

Methodologically the manuscript follows a logical sequence. Most results are well described and interpreted. However, It would be helpful to include a map of the study area, along with a clearer and more detailed description of the ‘Molecular Analysis,’ including the DNA extraction protocol, the procedure used for the individualization of the eggs analyzed, the ‘Control samples’ employed (Schistosoma haematobium, Schistosoma bovis, and Schistosoma haematobium/bovis), and the ‘Phylogenetic analysis’ (control and output, as well as the rationale for selecting the nucleotide substitution model). At present, these aspects are not entirely clear.

**Response:**

We appreciate the reviewer’s constructive suggestion. We have revised the Molecular Analysis subsection to include:

(i) the procedure for individualisation of schistosome eggs: “*The eggs were resuspended in 1 mL of 1 X Tris EDTA Buffer (pH = 8.0) in a petri dish. Under an inverted microscope, 10 individual eggs were removed from each sample with a Pasteur pipette, and each egg was placed into a separate 1.5 mL microcentrifuge tube containing 10 µL 1X Tris EDTA Buffer (pH 8.0). This ensured that DNA extraction was performed from a single egg, thereby avoiding mixed-template amplification.*” (lines 151–152),
(ii) reference to the published DNA extraction protocol used for extraction, “*DNA was extracted from each single egg using the Quick DNA Miniprep kit (Zymo Research, USA) following the manufacturer's instructions with some modifications which involved mechanical lysis (vortexing with glass beads), incubation with proteinase K, and elution of DNA as described elsewhere [24].”* (Line 156 – 157),
(iii) details of the modified primers used (Line 162 – 164 and Table S1),
(iv) the control samples employed: “positive *controls of S. mansoni, S. haematobium (DNA provided by the NIAID Schistosomiasis Resource Centre through BEI Resources, NIAID, NIH), and S. bovis [DNA provided by SCAN [26], as well as a negative (no template DNA) control, were used alongside each PCR to ensure specificity and accuracy of amplification.”* Line 167-170), and
(v) the phylogenetic analysis procedure. “*For phylogenetic analysis, a maximum likelihood (ML) tree was constructed using the Tamura-Nei model [27] with 1,000 bootstrap replications to assess branch support. The Tamura-Nei model was selected due to its ability to account for variable nucleotide substitution rates, providing a more accurate representation of evolutionary relationships. Its use therefore provides a more realistic estimation of evolutionary distances and improves the accuracy of maximum likelihood tree construction. including control sequences, outputs, and the rationale for selecting the nucleotide substitution model*” (Lines 198–202).

Results and discussion

This manuscript presents the results of a cross-sectional study investigating the prevalence of Schistosoma spp. in schoolchildren from two communities in Ghana. However, relying solely on the analysis of mtDNA sequences from a fragment of the cox1 gene may not provide sufficiently conclusive evidence to fully assess the prevalence of Schistosoma hybrids in the region. This work represents an excellent and much-needed opportunity to address the challenges related to the behaviour and evolution of the human and animal epidemiology of this disease in endemic areas. Such an endeavour requires the accurate identification of the parasitic forms involved, as well as of the intermediate hosts.

**Response:**

We thank the reviewer for this valuable comment. The focus of our study was on characterising hybrid *Schistosoma* infections directly in humans, specifically schoolchildren, as a means of understanding ongoing transmission. We note that this study only looked at the occurrence of the hybrids and not their transmission, and acknowledge that including intermediate host snails would provide complementary insights into transmission dynamics; however, this was beyond the scope of the present work. We have now clarified this in the discussion: “*Additionally, although our investigation focused on the occurrence of hybrids in humans, it is acknowledged that incorporating intermediate host snails and characterising the cercariae they transmit and their viability would provide complementary evidence of transmission dynamics*……….” (Lines 371 – 376) and highlighted that future studies integrating both definitive (human/animal) and intermediate (snail) hosts, alongside multilocus molecular analyses, would provide a more comprehensive understanding of hybrid schistosome transmission in endemic regions.
