# Supplementary material for: Prevalence of Schistosoma bovis and Schistosoma haematobium hybrids in endemic communities in Ghana
Source: PLoS One. 2026 Jan 7;21(1):e0339722. doi: 10.1371/journal.pone.0339722 (PMC12779034; doi:10.1371/journal.pone.0339722)
Supplement: Attachment — Submitted filename: Response to Reviewer Comments.docx [file pone.0339722.s006.docx]

Reviewers’ comment

1.My previous point about the unclear reason for the placement of samples M3, M5, M10 has not been appropriately addressed. The authors now list a large list of speculative reasons for the found pattern. However, many of them are not valid for a mitochondrial gene. For example:

“Beyond environmental adaptation, alternative explanations such as hybridization and introgression events with S. haematobium or other Schistosoma species could account for their distinct genetic profiles. These samples may have undergone genetic recombination or backcrossing, leading to their differentiation from other S. bovis isolates.” -> Hybridization and introgression do not occur for mitochondria since they are maternally inherited.

Overall, the phylogenetics are not discussed appropriately and are currently still confusing. Especially, one needs to consider the concerns raised by Reviewer 3 more carefully. I concur that the discussion of what can and cannot be said about hybridization based on mtDNA needs to be adjusted.

**Response:**

We thank the reviewer for the valuable feedback, which has helped us improve the clarity of our discussions.

To address the comments, we have limited the discussions on the introgression and hybridization to focus on the main results of the paper which showed the occurrence of *S. bovis* in samples identified as *S. haematobium*. The phylogenetic analysis were essentially to confirm the molecular findings. Thus, we have modified the title to reflect “**Prevalence of *Schistosoma bovis* and *Schistosoma haematobium* hybrids in endemic communities in Ghana”** removing the focus from the aspect of genetic diversity, which we agree needs further studies.

However, it is important to note that while mtDNA are primarily inherited maternally, there is some evidence of biparental inheritance in literature. Genome recombination plays a key role in generating genotypic diversity and facilitating adaptation and speciation after interspecies hybridization (Leducq et al., 2017; Schumer et al., 2014). Traditionally, this role has been considered mainly within the nuclear genome, as recombination is thought to be limited in organelles (such as mitochondria) due to their predominantly maternal inheritance (Barr et al., 2005). Thus, heteroplasmy – the co-occurrence of both parental mtDNA in a progeny – is rare and limited, but with some exceptions allowing for rare biparental recombination to take place (Breton & Stewart, 2015; Gyllensten et al., 1991; Kvist et al., 2003; Rokas et al., 2003; Xu et al., 2009). Thus, while mtDNA is generally maternally inherited in most animals, this can be affected by hybridization, leading to introgression and heteroplasmy. Further studies on this in schistosomes are warranted. While the phylogenetic analysis was essentially to confirm the molecular findings, a limitation of this study is the use of COX1 alone in the interpretation of the phylogenetic results. Thus, the interrelatedness of the hybrids should be interpreted with caution. Nonetheless, the findings from this study add to the growing evidence of genetic exchange between human and livestock schistosomes in West Africa (Borlase et al., 2021; Panzner & Boissier, 2021).

We have presented the above in the discussion, noting the importance of further studying heteroplasmy in schistosomes.

2.The discussion of statistics is still imprecise. If a test is not statistically significant then the wording needs to reflect that.

**Response:**

We thank the reviewer for highlighting the imprecise wording used in reporting statistically non-significant results.

We have revised the language in the Discussion to accurately reflect those differences in infection intensity by location (Ga South vs. Birim North), age group, and gender were not statistically significant.

The sentence previously stating that “higher intensity infections were observed among older age groups” now specifies that “although higher proportions were observed in the 13–17 age group in Ga South, the differences were not statistically significant”

Similarly, we have clarified that the higher infection rates in males across both districts did not reach statistical significance and should be interpreted cautiously.

These changes enhance the statistical rigor of our discussion and align with standard scientific reporting practices.

We hope these revisions satisfactorily address the reviewer’s concerns and improve the overall clarity and scientific integrity of the manuscript.
